# Poisonous milk and sinful mothers: the changing meaning of breastfeeding in the wake of the HIV epidemic in Addis Ababa, Ethiopia

**DOI:** 10.1186/1746-4358-5-12

**Published:** 2010-10-26

**Authors:** Absera T Koricho, Karen Marie Moland, Astrid Blystad

**Affiliations:** 1 Department of Public Health Officers, Hawassa University, Hawassa, Ethiopia; 2 Department of Health and Social Sciences, Bergen University College, Bergen, Norway; 3 Department of Public Health and Primary Health Care/Centre for International Health, University of Bergen, Norway

## Abstract

**Background:**

Breastfeeding remains normative and vital for child survival in the developing world. However, knowledge of the risk of Human Immunodeficiency Virus (HIV) transmission through breastfeeding has brought to attention the controversy of whether breastfeeding can be safely practiced by HIV positive mothers. Prevention of mother to child transmission (PMTCT) programs provide prevention services to HIV positive mothers including infant feeding counseling based on international guidelines. This study aimed at exploring infant feeding choices and how breastfeeding and the risk of HIV transmission through breastfeeding was interpreted among HIV positive mothers and their counselors in PMTCT programs in Addis Ababa, Ethiopia.

**Methods:**

The study was conducted in the PMTCT clinics in two governmental hospitals in Addis Ababa, Ethiopia, using qualitative interviews and participant observation. Twenty two HIV positive mothers and ten health professionals working in PMTCT clinics were interviewed.

**Results:**

The study revealed that HIV positive mothers have developed an immense fear of breast milk which is out of proportion compared to the evidence of risk of transmission documented. The fear is expressed through avoidance of breastfeeding or, if no other choice is available, through an intense unease with the breastfeeding situation, and through expressions of sin, guilt, blame and regret. Health professionals working in the PMTCT programs seemed to largely share the fear of HIV positive mother's breast milk, and their anxiety was reflected in the counseling services they provided. Formula feeding was the preferred infant feeding method, and was chosen also by HIV positive women who had to beg in the streets for survival.

**Conclusions:**

The fear of breast milk that seems to have developed among counselors and HIV positive mothers in the wake of the HIV epidemic may challenge a well established breastfeeding culture and calls for public health action. Based on strong evidence of the risks when infants are not exclusively breastfed, there is a great need to protect breastfeeding from pressures of replacement feeding and to promote exclusive breastfeeding as the best infant feeding option for HIV positive and HIV negative mothers alike.

## Background

The evidence of the risk of Human Immunodeficiency Virus (HIV) transmission through breastfeeding has caused major dilemmas in public health and has created a lot of uncertainty among infant feeding counselors and HIV positive women, as well as in the population at large [1-3]. The knowledge of the fundamental significance of breastfeeding, particularly in low income contexts where mothers in most cases have no safe and affordable alternatives to breastfeeding, has been a most serious challenge in the attempts to Prevent Mother to Child Transmission (PMTCT) of HIV [2,4].

Without intervention 30-45% of all infants born to HIV positive mothers will be infected and 10-20% will be infected through breastfeeding [3]. However, great efforts have been put into making breastfeeding safer particularly in resource-poor settings. Research has documented that it is mixed breastfeeding - the combination of breastfeeding with other nutrients - that implies the highest risk of HIV transmission. By contrast, exclusive breastfeeding (EBF) - breastfeeding without any other nutrient fed to the infant - is almost as safe as replacement feeding in terms of HIV transmission [5-7], and safer in terms of HIV free survival. A study conducted in South Africa, showed that exclusive breastfeeding carried a transmission rate of 4% from six weeks after birth up to six months [7], hence approaching the transmission rate in high income contexts estimated to be 2%. A major challenge has been to ensure that breastfeeding is practiced exclusively. The same study importantly documented that the cumulative 3 month mortality rate was significantly lower in exclusively breastfed infants than in replacement fed infants (6.1% versus 15.1%) [7].

Infant feeding counseling in PMTCT programs in Ethiopia as elsewhere in sub-Saharan Africa has been based on the 2001 World Health Organization (WHO) infant feeding guidelines which promote replacement feeding as the best option if acceptable, feasible, affordable, sustainable and safe (AFASS) [2,4,8,9]. The guideline has been criticized for its lack of local relevance and the recommended infant feeding options. Exclusive breastfeeding and exclusive replacement feeding (infant formula or animal milk) have proven to be hard to implement for women enrolled in PMTCT programs. Practical, economic, social, psychological and cultural challenges are encountered in connection with both exclusive breastfeeding and replacement feeding [4,8,9]. Some of the key challenges have been linked to poor counseling or poor training of counselors [4], to customary infant feeding practices and to social expectations to breastfeed. In sub-Saharan Africa early mixed feeding and prolonged breastfeeding, i.e. prolonged mixed feeding, is the norm [5-7] and fundamentally challenges the PMTCT concept. The social expectations to breastfeed are strong and the risk of HIV positive status disclosure if practicing replacement feeding has been experienced as critical. This has led many mothers to combine breastfeeding and replacement feeding as the situation requires [9,10].

The increase in infant deaths linked to replacement feeding, also in cases of free distribution of infant formula [11], and the increasing evidence that exclusive breastfeeding can be a safe option also for HIV positive mothers have led to a shift in the guidelines of the WHO. The updated guidelines from 2007 and 2009 promote and recommend exclusive breastfeeding for six months for all HIV positive mothers if replacement feeding is not AFASS [12,13]. Hence, replacement feeding is recommended only as the second choice and if AFASS. Although the new recommendations had not been implemented in Ethiopia at the time of the study, counselors were aware of the situation and many were concerned and uneasy about the promotion of exclusive breastfeeding as a safe infant feeding option for HIV positive mothers.

The present article explores how breastfeeding and the risk of HIV transmission through breastfeeding is interpreted by HIV positive mothers and counselors in PMTCT programs in Addis Ababa, and how this is expressed through infant feeding practices.

### Study setting

The study was carried out in Addis Ababa, Ethiopia, from June to August in 2007. The population of Addis Ababa city is estimated at 3,059,000 people [14]. A single point HIV prevalence estimate in June 2007 suggested HIV prevalence in Addis Ababa of 7.5% (6% in the male and 8.9% in the female population). In 2003, the antenatal care (ANC) estimate of the HIV prevalence among pregnant women in the city was 12.4%.

The first National PMTCT guidelines were published in 2001 by the Ministry of Health, Ethiopia [15]. Today PMTCT services are offered at health centres and hospitals in all sub-cities in Addis Ababa and are slowly expanding into the rural parts of the country [16].

The study settings were two governmental hospitals situated in the city of Addis Ababa. The two hospitals were among the first to offer PMTCT services in the country starting in 2003 and in 2004 respectively. The first 'infant feeding and HIV follow-up clinic' was established in one of the hospitals in 2004, and three nurses had been trained and were providing PMTCT services including infant feeding counseling at the time of the fieldwork in 2007. In the second hospital, there was no specific 'infant feeding and HIV follow-up clinic', and the PMTCT services were more fragmented. Pre-partum counseling was provided in the antenatal clinic and the post-partum follow-up was done from the pediatric Antiretroviral Treatment (ART) clinic by staff with limited training in PMTCT and infant feeding counseling.

At the time of the study, PMTCT services in the two hospitals included a standard package of pre- and post-test counseling, infant feeding counseling during pregnancy and a single dose of prophylactic antiretroviral drug (nevirapine) during labour for the mother and immediately after birth for the infant. The routine follow up at six weeks after delivery included growth monitoring, infant feeding counseling and prophylactic antibiotic (sulfamethaxazole-trimethoprim) to prevent bacterial infections in the baby. Eligible mothers were referred to adult ART clinics for treatment.

## Methods

### Study design

This is a qualitative interpretive study employing qualitative triangulation methods. Data were collected through qualitative interviews and participant observation.

### Data collection

Thirty two in-depth qualitative interviews with HIV positive mothers, nurse counselors and physicians were conducted. Four nurses, two from each hospital were recruited as research assistants. They recruited HIV positive infant feeding mothers to participate in the study, sought their informed consent and informed them about the principles of research ethics including voluntary participation, the rights of withdrawal, anonymity and confidentiality. With one exception, all the mothers approached agreed to participate in the study. Three different interview guides were used for the three categories of informants (HIV positive mothers who breastfed their infants, HIV positive mothers who replacement fed their infants and health professionals). The interview guides included open ended questions with a set of potential questions for follow up/probing. Twenty two HIV positive infant feeding women were recruited. The number of informants was not predetermined, but was based on data saturation where the major themes were increasingly repeated. All health professionals in both hospitals who were linked to the PMTCT programme; eight nurses and two medical doctors were interviewed. One doctor and five nurses were interviewed from one of the hospitals and the rest, one doctor and three nurses, were interviewed from the other hospital.

Interview guides were developed for the two major groups of informants. The questions focused on the experiences of patients and health care workers in the PMTCT program. All interviews were conducted by the first author in the national language, Amharic, and were tape-recorded. The audio data were transcribed by professional transcribers. To maintain confidentiality of the information and to avoid any possibility of identification of the informants, caution was taken to avoid any information that could reveal the identity of the informants. The transcripts were checked against the original recordings by the first author. The transcribed data was then translated to English, by the first author who tried to render a translation that was as close as possible to the original language. Special attention was paid to retain the cultural dimension inherent in the informants' expressions. To evaluate the equivalence between the original and English version, the transcribed text was checked against the original recordings throughout the research process.

### Data analysis

The procedure of analysis employed draws upon the principles of thematic analysis as described by Pope and Mays [17]. We carried out the whole data analysis process manually. The analysis involved reading and re-reading all the data sets in order to identify an initial set of themes. The data were then systematically searched for recurring themes, as well as for views or experiences that were different or that contradicted the main emerging patterns. Following this initial process, the text was coded and categorized by the first author in close collaboration with the third author. Coding was carried out manually. In merging the codes into broader categories and themes, the researchers continuously ensured that all the major content was accounted for.

To supplement the interview data, the first author, who is trained as a health officer, participated in the daily work at the two clinics and observed routines, procedures and patient-health worker interaction. Field notes linked to particular observations and to questions and reflections emerging during the field work period were noted down in some detail. The field notes were employed to increase the contextual understanding of the topic studied.

All the raw material as well as the coded material were kept in a secure place to ensure safety and confidentiality. The raw audio and the soft copy of the data were kept on a personal computer of the first author protected by a password. Research permit and ethical clearance was obtained through the Regional Health Bureau of Addis Ababa, and a letter of permission was granted from the respective hospitals. Participants were financially compensated for transportation and lost time based on the existing practice of the health institutions.

The data collection was limited to governmental hospitals with free services where the majority of the patients have low socio-economic status. Home visits to the mothers which could have added important information were not performed due to time constraints.

## Results

### Demographic information

The mothers came from different parts of Addis Ababa. The great majority belonged to the Orthodox Church (18/22) while two belonged to the Protestant congregation and two were Muslims. Their age ranged from 22 to 39 years, and their level of education varied between completed high school (2/22) and no formal education (2/22). Four had a paid job (4/22). Sixteen were married, five were divorced and one was single. The five divorcees reported that their husbands had left them after they disclosed their HIV status. The majority of mothers (19/22) had disclosed their HIV status to at least one person. There was no difference between the disclosure status of the mothers who breastfed and the mothers who did not breastfeed. Only two mothers knew their HIV status before their last pregnancy. Slightly more than half of the interviewed HIV positive mothers (12 of the 22) already were taking ARV for treatment.

The health professionals interviewed were between 25 and 40 years old and had three months to more than three years of experience in the PMTCT program. The majority were women (7/10).

### Poisonous milk and sinful mothers

In the following result section we present the perceptions and experiences of mothers struggling to adhere to their infant feeding choice, and the perceptions of counsellors who seem to counsel mothers only with one aim in mind: to secure an HIV negative status of the child. The major themes that run through the findings are bad milk and bad mothers, and the interconnection between the two.

#### Abhorrence

The interviews with the HIV positive mothers revealed a strong fear of breast milk.

The knowledge of the possibility of infecting their baby with HIV through their breast milk was experienced as extremely difficult to live with. One mother who was convinced that her child had already been infected through her milk said:

"I was sure that I was going to continue breastfeeding for six months, but knowing that I am poisoning my child can't give me peace of mind. You feel like you are a criminal doing something bad to your own child. That was how breastfeeding was for me, and now I don't even want to think that I breastfed for one second." (Mother #1)

The concept that the milk of an HIV positive mother was like poison to the baby and could kill her/him made the breastfeeding situation almost unbearable. Some mothers talked about the loathing they felt for their own body as infectious and dirty and not being able to nourish the newborn with safe and clean mother's milk. This repugnance sometimes had physical manifestations as the following quote from a young mother illustrates:

"Every time the baby was sucking my breasts I felt like throwing up." (Mother #2)

With breastfeeding as a central symbol of motherhood, the experience of bad breast milk was closely associated with the experience of bad motherhood.

#### Guilt and regret

Breastfeeding was experienced by many mothers as an act that was morally wrong or bad. Some talked about this 'wrong-doing' as a sin and thus as an offence against God. An HIV negative child after breastfeeding was only possible through God's mercy:

"I now regret what I did, but God is merciful and he didn't see my sin. She (the nurse) told me that he (the baby) is negative." (Mother #3)

Others talked about breastfeeding as a crime that should have been punishable in court since it inflicted harm on another human being:

"I cannot believe I was breastfeeding my child knowing that I have the virus in my breast milk. If you were a judge, you might have sent me to jail. I almost killed him." (Mother #4)

The idea of breastfeeding as a sin if practiced by HIV positive mothers was closely associated with the link to HIV. Most of the HIV positive mothers who breastfed their infants felt sinful. The experience left a challenging reminder of their infection and their situation. They expressed the helplessness that they felt while breastfeeding and that the experience was painful because they unable to avoid it. A mother who had breastfed her child for four months said:

"I don't even want to talk about those four months that I breastfed my child. I was cursing myself. Every time the breastfeeding reminded me that I am HIV positive and that my breast milk was filled with the virus. It is my sin, and I am the one who is being punished, but transferring it to my child is also a sin. What else can it be?" (Mother #5)

Whether the exposure of the baby to the HIV virus through breastfeeding was defined as a sin or as a crime, the responsibility for the HIV status of the baby remained with the mother. Hence strong feelings of guilt or self-blame emerged in the interviews:

"I am very lucky that my child is HIV negative. If he were positive, I know what I would have done, I would have killed myself. But thanks to God, He saved me and my baby." (Mother #3)

For the informants who had breastfed their babies the feelings of guilt were linked to a regret that was often powerfully revealed in their accounts. One mother said:

"I thought there was no choice other than breastfeeding. If she (the counsellor) had told me that I could buy formula milk from the beginning, I would have done that long ago." (Mother #6)

Another mother said:

"If God gives me a second chance to live the last two months over again, I would rather stop eating and buy tin milk [infant formula] for my child". (Mother #7)

The emotional strain experienced during the breastfeeding period implied that many women decided to discontinue breastfeeding at a very early point.

#### Fear of breast milk

Others were so afraid of breastfeeding that they chose to replacement feed their babies with either infant formula or cow's milk. Replacement feeding attempts were made despite the often low chances of succeeding in terms of adhering to this infant feeding method which was both extremely costly and highly uncustomary.

Very few mothers who were replacement feeding their babies (8/22) had a permanent income. A few (two out of eight) opted for formula feeding even though they simply had no money to buy the infant formula. For most women however, the formula milk would be available for the first two to three months only. Some of these mothers were begging in the street in order to get money to buy replacement milk, commonly cow's milk. A 28 year old HIV positive mother said:

"I know Ethiopian people are good and they won't let you down. Especially if you tell them that you have the virus and a baby. You will not suffer that much. You will get what is needed for that day. The next day you go out for the same thing. Of course, it is hard to be out there and beg. But what can you do? Kill your baby?" (Mother #8)

#### The 'naturalness' of breastfeeding

In a context where breastfeeding is perceived as the only natural way of feeding an infant, women who chose not to breastfeed were confronted with the expectations from kin and others that a woman should breastfeed. For mothers who had not disclosed their HIV positive status, the need to conceal their status and also avoid breastfeeding added to their emotional strain as the following example illustrates:

"It was my daughter's baptism. I was not breastfeeding my baby. She started crying and I gave her a bottle. People around asked me why I was not giving her my breasts. I told them I was bottle feeding her because my breasts did not have enough milk. My elder sister stood up immediately, came with warm water, and started massaging my breasts. She was trying to express my breast milk. I could not try to stop her. Then she told me to put my nipples in to my baby's mouth. I had nothing to say, and had to do what she told me to do. Since my breast was new for the baby, she did not take it immediately. Instead, she started playing with it. I told them she does not like my breasts. I was praying to God so that she would not be able to suck." (Mother #9)

### Saving the child from HIV: the counselors' view

#### Communicating choice

The mothers' fear of breastfeeding was clearly linked to the counseling they received in connection with pregnancy and birth. During most of the counseling sessions the information about the risk of HIV transmission through breastfeeding came across very powerfully, and strongly discouraged HIV positive mothers from breastfeeding. One of the informants who had decided to avoid breastfeeding by all means and who fed her baby replacement milk recalled how the counseling advice created a fear that affected her choice of infant feeding method:

"When the nurse told me that I have HIV in my breasts, I became so scared of breastfeeding. I didn't have savings to buy tinned milk, but I couldn't feed my child my breast milk which is filled with my virus." (Mother #10)

The fear of the HIV positive breast milk was indeed found to be strongly present also among the nurse counselors, and the manner in which they communicated the information about the infant feeding options was often emotional and reflected their own fear. One mother recalled:

"The nurse almost screamed at me saying 'Why are you going to breastfeed your baby? You took nevirapine and you have a great chance of having an HIV negative baby. Are you going to give your baby your disease?' At that point I was happy that the delivery went well, but I couldn't bear the thought that I was going to infect my baby." (Mother #11)

The counselors' often did not introduce all the infant feeding options described by WHO because of their fear of HIV transmission from mother to child through breast milk. They would merely present avoidance of all breastfeeding as the following quote illustrates:

"The nurse told me that I have HIV. Since I knew nothing about the virus, I asked her what I should do. She told me to buy cow's milk and I started feeding my child cow's milk. I have told no one that I have HIV." (Mother #12)

#### Promoting replacement feeding

The nurse counselors had a negative attitude to breastfeeding by HIV positive women. Eight out of ten health professionals in the study would strongly defend their promotion of replacement feeding and their neglect or silence about exclusive breastfeeding as another recommended infant feeding option for HIV positive mothers. One counselor stated:

"Why should an HIV positive mother breastfeed when it is known that HIV can be transmitted through her milk? I mean, imagine that baby with HIV coming to our clinic to get ART from his young age. I cannot understand that. Therefore I would say HIV positive mothers should be strong enough not to breastfeed at all. That is the only choice if we want an HIV-free generation." (Health professional #1)

Many counselors would feel great satisfaction if their clients abstained from breastfeeding, since they were convinced that this effort would "save the child from HIV infection" as illustrated in the statement:

"There are very few mothers who practice breastfeeding here [in the clinic] and we all are very happy about that." (Health professional #2)

At the time of the study, there were rumors about the upcoming revision of the WHO HIV and infant feeding guidelines and its emphasis on breastfeeding as the first choice also among HIV positive women. This was met with concern and unease by many of the counselors. As one nurse counselor working in one of the two hospitals explained:

"I really hope that the new recommendation is only for discussion; not for actual practice. How can we tell these mothers? They have been told repeatedly about the risk of HIV transmission through breastfeeding, and now all of a sudden breastfeeding is 'good' again." (Health professional #3)

#### Diverging counseling routines

Interestingly the opinions and infant feeding counseling routines seemed to differ to a great extent between the two hospitals included in the study. While the counselors at one hospital unanimously argued against breastfeeding, several counselors at the other hospital emphasized that an assessment of the AFASS criteria (acceptability, feasibility, affordability, sustainability and safety) was decisive for their infant feeding counseling of each mother. One of the counselors explained that he had never met a single mother who met the AFASS criteria, so the counselors would routinely counsel HIV positive women on exclusive breastfeeding only. He explained:

"Let us say that a mother comes and she is poor looking and doesn't have phone number to contact. For mothers like her, we do not even tell the existence of another choice than breastfeeding. When the time comes (when the baby is six months old) we will explain to her about the risk of continuing breastfeeding." (Health professional #4)

The different infant feeding counseling practices thus seemed to partly follow the respective institutions. Making an assessment of the individual woman's options was influenced by the preferences of individual counselors and only to a very limited extent followed the guidelines. Infant feeding counseling was dominated by the concern to save the child from HIV more than working towards infant survival. With this aim in mind, the majority of the counselors ignored the content of the AFASS criteria.

## Discussion

The study revealed that the choice of infant feeding method among HIV positive mothers in Addis Ababa was strongly influenced by their fear of breast milk. Most of the mothers and nurse counselors interviewed dealt with infant feeding in a manner that was completely ruled by the knowledge that HIV can be transmitted through breastfeeding. With a couple of exceptions, concerns about the AFASS of replacement feeding did not surface in a routine manner in infant feeding counseling sessions. We have seen above that the HIV positive mothers in our study who breastfed without knowing the risk of HIV transmission deeply regretted their acts. Many of these mothers would in fact hold health professionals responsible for not informing them that they "were giving poison to their infants." Similar findings have been described in a qualitative study from South Africa where an HIV positive mother described her breastfeeding experience by saying ". . . I thought I was breastfeeding, but I was breast-poisoning" [18].

Both mothers' and nurse counselors' accounts revealed a lack of knowledge of the actual risk of HIV transmission, and of the potential threat to child survival linked to not breastfeeding. Their deliberations were moreover largely emotional, and in the context of strong religious tradition, they seemed to be colored more by the symbols of sin and evil associated with HIV transmission than by medical knowledge related to child survival. The moral condemnation of themselves as carriers and transmitters of HIV infection and hence of immorality, must also be understood in a poverty context where motherhood commonly is seen as the purpose of womanhood. Breastfeeding in the context of PMTCT was not promoted for its nourishing and life enhancing qualities, but was rather discouraged as poisonous and life threatening. As HIV was perceived as a deadly virus to which there is no cure, breastfeeding became almost equivalent to knowingly killing the baby.

Earlier qualitative studies from Tanzania and Ethiopia conducted among HIV positive mothers who were breastfeeding their infants have described the pain and fear associated with breastfeeding [9,19]. Furthermore, Leshabari and colleagues highlighted the tension between the messages presented during the counseling sessions and mothers' desire to practice breastfeeding [9]. Moland and Blystad also demonstrated that knowledge about infant feeding and HIV was very confused among both HIV positive mothers and their counselors [19]. The confusion was linked to the distorted assumptions about the risk of transmission that HIV positive mothers and their counselors shared. However, these studies have not identified to the same extent as the current study the unease and guilt related to the breast feeding experience itself and the growing fear linked with breast milk. Based on the findings of this study, we argue that it seems to be the fear of breast milk rather than a medical and economic risk assessment that is the main driving force behind HIV positive mothers' infant feeding choices and the advice given by the nurse counselors.

We have seen that the serious implication of the fear of mothers' milk is that many HIV positive mothers choose to replacement feed their babies. None of the mothers in the present study were likely to fulfill the WHO-AFASS criteria for replacement feeding. Only a couple had a permanent income, and the ones who opted for replacement feeding had to rely on passers-by to contribute to sustain the purchase of expensive infant formula. Other studies have similarly indicated that HIV positive mothers who live in resource-limited settings chose to replacement feed their babies without fulfilling the AFASS criteria [19]. Similar studies link the choice of formula feeding among their study participants to the free provision of formula feeding as well as to poor counseling. It is not unlikely that fear of breast milk underlies both the provision of free formula, and the distorted messages regarding breast milk that the authors describe [19].

The information base of the nurses emerged as very limited and their advice to the mothers was largely based on either hospital policy or personal preference. In fact, the little training received by some of the nurses seemed to largely contribute to the notion that breast milk kills and should thus be avoided. The fundamental knowledge that the baby will be far more likely to suffer and die if given infant formula than if breastfed was not brought up by any of the mothers or counsellors.

The nurse counselors' role in the spread of the fear of mothers' milk emerged strongly in the present study findings. Studies from Tanzania and South Africa have similarly revealed the substantial influence of nurse counsellors over HIV positive mothers' infant feeding choices [4,18]. The findings in the present study have revealed a high level of fear and scepticism among counsellors to HIV positive women's breast milk - a fear naturally transferred to their clients. Indeed they seemed to define their role in infant feeding counseling as one of preventing HIV transmission from mother to child rather than promoting child survival through exclusive breastfeeding.

## Conclusions

The reduction in breastfeeding in the last two decades, especially in some settings, has been attributed to AIDS, and the decline in the practice has been called "the greatest nutritional crisis" [20]. The major reason for the decline is said to be the uncertainty related to HIV and breastfeeding. The findings of the present study, which indicate that many HIV positive mothers choose not to breastfeed despite unfavourable economic and social conditions for replacement feeding, is a strong manifestation of this process.

The prevailing WHO guidelines at the time of fieldwork (the 2001 guideline on HIV and infant feeding) was about to be modified in the direction of placing a greater emphasis on exclusive breastfeeding by all HIV infected mothers [2]. Based on our findings from the present study, we suggest that the promotion of breastfeeding among HIV positive women as advised in the revised 2009 guidelines will face enormous challenges in its way forward. However, based on the strong evidence of the risks when infants are not exclusively breastfed, even in babies of HIV positive mothers, there is a great need to join forces in the struggle to protect breastfeeding from pressures of replacement feeding, and to promote exclusive breastfeeding as the best infant feeding option for HIV positive and HIV negative mothers alike.

## Competing interests

The authors declare that they have no competing interests.

## Authors' contributions

The article is based on AK's master's thesis in International health. She designed the study and analyzed the data in collaboration with her supervisor AB. She also collected all the data, and wrote the first draft of the article. AB and KMM contributed substantially to the writing process. All authors read and approved the final manuscript.
